# Development of in-house ELISA based on recombinant gag proteins of small ruminant lentiviruses isolated from goats in Thailand

**DOI:** 10.1038/s41598-024-54360-x

**Published:** 2024-02-13

**Authors:** Tatchapon Mongkonwattanaporn, Preeda Lertwatcharasarakul, Theera Rukkwamsuk

**Affiliations:** 1 Department of Large Animal and Wildlife Clinical Sciences, Faculty of Veterinary Medicine, Kasetsart University, Malaiman Road, Kamphaeng Saen, Nakhon Pathom, 73140 Thailand; 2 Department of Pathology, Faculty of Veterinary Medicine, Kasetsart University, Malaiman Road, Kamphaeng Saen, Nakhon Pathom, 73140 Thailand

**Keywords:** ELISA, Phylogeny, Recombinant protein, Small ruminants, Immunology, Molecular biology

## Abstract

Small ruminant lentiviruses (SRLVs), are grouped in *Retroviridae* family, remain a significant loss in the small ruminant husbandry. As a result of unavailability of vaccine and effective treatment, the diagnosis plays a crucial role for the control of SRLV infection. However, the major challenge of diagnosis of SRLV infection is the genetic and antigenic variability of the viruses that can lead to a failure in serological detection. This study investigated the circulating strains of the viruses in goats in Thailand and an in-house ELISA was developed. The coding sequences for gag protein were optimized, synthesized, and expressed in *Escherichia coli* for increasing the sensitivity of ELISA test. A total of 365 serum samples were examined against the recombinant protein in an in-house ELISA. The results showed that the recombinant gag achieves 96.67% sensitivity and 93.18% specificity as compared with the commercially available ELISA test kit.

## Introduction

Small ruminant lentiviruses (SRLVs) are classified among the genus Lentivirus in *Retroviridae* family. These viruses can induce persistent infection in sheep and goats. The SRLV genome comprise of two linear positive-sense single-stranded RNA subunits, which compose of *gag* (group-specific antigens), *pol* (polymerase), *env* (envelope), and long terminal repeats (*LTRs*) genes in addition to a group of regulatory genes. Both single-stranded RNA subunits contain 3 main genes: *gag* and *env* codify structural protein while *pol* codified enzymatic protein^1–3^. SRLV can be categorized into 5 groups: A, B, C, D and E. While genotype A and B, occasionally introduced to as Maedi-visna virus-like and Caprine arthritis encephalitis virus-like, respectively, are widely distributed, whereas genotypes C and E are found in the confined areas. Genotype D was described only *pol* sequences while phylogenetic analysis on *gag* sequences of genotype D was classified these sequences into group A^3,4^. Previous studies have provided evidence supporting the inter-species transmission of viruses between goats and sheep^5–8^.

The SRLV infection has been reported worldwide with high variation of prevalence, including in Egypt (8.52%)^9^, Mexico (0.4%)^10^, Southern Spain (23.22%)^11^, India (19.58%)^12^, Japan (10.0%)^13^, South Korea (2.73%)^14^, and Thailand (2.60%)^15^.

The major route of SRLV transmission is through infected colostrum or milk ingestion or inhalation of the respiratory secretion^16^. Once SRLVs are transmitted, they preferably infect macrophages and monocytes, where they incorporate their genome into the host genome. By means of the circulation, precursor cells in bone marrow are infected by the circulating infected macrophages and monocytes leading to a life-long infection^2^. Most infected animals show no clinical manifestation and SRLV infection is mostly in the form of subclinical infection^7^. However, some infected animals can exhibit pathological manifestations like arthritis, encephalitis, pneumonia, and mastitis^17,18^.

Currently, there is no effective vaccines or medication available in the prevention or treatment of SRLV infection. Accordingly, the precise diagnosis has an important role to avoid viral dissemination. The most common methods of routine diagnosis are the enzyme-linked immunosorbent assay (ELISA) and the agar gel immunodiffusion (AGID) test. Nonetheless, the delayed seroconversion in some infected animals can result in the false negative from the ELISA^19,20^.

In addition to serological tests, molecular methods such as PCR were developed for the detection of the viral nucleic acid. However, SRLV infection diagnosis by using molecular methods may be unreliable because of the low quantity of RNA or provirus in the infected animals and the high genetic variability of the viruses^1,21^. Thus, knowledge about circulating strains of the virus is crucial for disease diagnosis. Nonetheless, there are several reports showed that the combination of PCR and serologic testing is crucial to the development of an effective control program^22,23^. For the serological methods, the antibody titers can vary throughout the course of infection, so the antibody detection depends upon the sensitivity of the test. Due to the intermittent appearance of antibody, the detection of the viral nucleic acid, such as PCR, prior to seroconversion is essential for a more accurate diagnosis^24^.

In general, the conserved gene is a favorable candidate for diagnosis. Both *gag* and *pol* genes are relatively preserved in SRLV genome, making them optimal sites for PCR primers design. Gag proteins tended to have higher antigenicity. Although SRLV infections have been reported in small ruminants in Thailand for a long time, the studies about the molecular characterization of this virus in goats are limited. In this study, the phylogenetic tree of the *gag* gene of SRLVs was developed from the sequences obtained from circulating viruses in Thailand and the in-house ELISA based on the results of local strain of SRLVs was developed.

## Materials and methods

This study was approved by the Kasetsart University’s Institutional Animal Care and Use Committee. The approved number is ACKU64-VET-007. The authors confirmed that all experiments were performed in accordance with relevant guidelines and regulations of the animal care and use under the Animals for Scientific Purposes Act, B.E. 2558 (2015).

### Sample collection and proviral DNA extraction

The samples were taken from whole blood, spleen, and brain of CAE suspected goat. DNA was isolated using a commercially available DNA extraction kit (FavorPrep™ Tissue Genomic DNA Extraction Mini Kit, FAVORGEN Biotech Corporation, Taiwan) according to the manufacturer’s manual. Afterward, nucleic acids were subsequently quantified and verified by the NANODROP 2000 spectrophotometer (ThermoFisher Scientific, USA) and stored at  − 20 °C until they were used.

### Nested PCR primers

Nested PCR was performed for amplification of proviral DNA from the extracted DNA using the specific primers of the *gag* gene of SRLVs^25^. *ACTB* gene was amplified by using specific primers^26^ to confirm the quality of DNA samples (Table 1).

**Table 1:** List of the primers used in the study.

Primer name	Sequence	Round
Beta FW	TGCCCTGAGGCTCTCTTCCA	*ACTB* gene
Beta RV	TGCGGATGTCGACGTCACA	*ACTB* gene
CAE FW0	AACTGAAACTTCGGGGACGCCTG	1
CAE FW1	AAGGTAAGTGACTCTGCTGTACGC	1
CAE FW2	TGGTGAGTCTAGATAGAGACATGG	2
CAE RV0	GTTATCTCGTCCTAATACTTCTACTGG	1
CAE RV1	TTTTTCTCCTTCTACTATTCCYCC	1
CAE RV2	GGACGGCACCACACGTAKCCC	2

### General protocol for nested PCR and DNA sequencing

The DNA samples were amplified a fragment of the *gag* gene by using nested PCR. The target fragment amplifications were carried out with a final volume of 20 μL containing 0.4 U of DNA polymerase (Phusion™ High–Fidelity DNA Polymerase), 200 μM of each dNTP, and 0.5 μM of each primer. The following PCR conditions were applied: initial denaturation at 98 °C for 1 min, 40 cycles of denaturation at 98 °C for 15 s, annealing at 58 °C for 30 s, and extension at 72 °C for 1 min, and a final extension at 72 °C for 10 min. For the second round, 2 μL of product from the first round was added as a DNA template, and the nested round of PCRs were performed in the same condition. Then, products were visualized on 1.5% agarose gel containing RedSafe™ Nucleic Acid Staining Solution (iNtRON Biotechnology, Gyeonggi-do, Korea). The nested PCR positive samples in agarose gel were purified by using FavorPrep™ GEL/PCR Purification Kit (FAVORGEN Biotech Corporation, Taiwan) and sequenced using the Sanger sequencing method (Sequencer ABI3730XL, Bionics Co., Ltd., South Korea).

### Multiple sequence alignment and phylogenetic tree construction

Multiple alignments of the partial *gag* gene were generated. Phylogenetic construction was accomplished by aligning the newly obtained sequences with the GenBank reference sequences using the Neighbour-Joining (NJ method) with the Tamura-Nei gamma distance achieved in MEGA version X^27^.

### Expression of recombinant protein

The partial part of the *gag* gene of 2 genotypes of SRLVs (KU-003 and KU-009) was selected, optimized, synthesized, and cloned into pET-28b ( +) vectors at a specific restriction site (HindIII and NotI) and transformed into BL21 (DE3) competent *E. coli*. The transformed *E. coli* was spread on the 25 μg/ml kanamycin supplemented LB agar and incubated at 37 °C. Then, a colony was picked up and grown with 180 rpm shake in kanamycin supplemented LB broth at 37 °C. The culture was streaked on LB agar and incubated overnight at 37 °C. The single colony on LB media were confirmed the existence of *gag* gene by using PCR. The colony which contains the target gene was selected and subsequently cultivated in kanamycin-supplemented LB broth at 37 °C with agitation at 180 rpm overnight. Afterwards, the 100 μl of overnight culture were added into 5 ml of the same antibiotic supplemented LB broth and shaken for 3 h (or until an O.D. of 0.5 was reached at 600 nm). The induction of protein production was done using IPTG to a final concentration of 0.1 μM and incubated with agitation at 180 rpm for 4 h at 37 °C. The cells were taken using 10000 × g centrifugation for 5 min at 4 °C and underwent protein purification.

### Recombinant protein purification

The recombinant protein tagged with histidine was purified by an anti-His-tag immune-affinity chromatography (ÄKTA™ start protein purification system, GE Healthcare Bio-Sciences AB) in accordance with the manufacturer’s manual.

### Western blot analysis of recombinant protein

The western blotting was accomplished to figure out the immunoreactivities of the recombinant protein. After SDS-PAGE, the recombinant protein was transferred to a nitrocellulose membrane. The protein-transferred-membrane was blocked using blocking buffer (10% horse serum and 5% skimmed milk in PBS) for 60 min at 37 °C. Then, the membrane was incubated with positive SRLV goat serum diluted 1: 500 in primary antibody buffer (10% horse serum, 5% skimmed milk, and 10% *E. coli* lysate) for 1 h at 37 °C. After 3 times of washing process with 0.05% Tween 20 in PBS for 3 min, the membrane was incubated with the rabbit anti-goat IgG (H + L) (KPL Peroxidase Labeled Affinity Purified Antibody to Goat IgG (H + L) [Rabbit]) diluted 1: 1000 in secondary antibody buffer (10% horse serum and 1% skimmed milk in PBS) at 37 °C for 60 min. Then, the membrane was washed 3 times for 5 min. The recombinant protein was detected using TMB substrate as chromogenic substrate.

### Samples for enzyme-linked immunosorbent assay (ELISA) development

In this study, the serum samples were obtained from 365 goats. All samples were divided by using a commercial ELISA test kit (IDEXX CAEV/MVV total Ab test®, IDEXX Laboratories, Inc., Maine, USA) into 2 groups: the positive samples and negative samples.

### ELISA general protocol

100 μl of 12.5 μg/ml SRLV-gag protein in coating buffer (1.5% Na_2_CO_3_ and 2.93% NaHCO_3_ in distilled water) was coated into 96-well ELISA plates (Nunc-Immuno™ MicroWell™ 96 well solid plates) at 37 °C for 1 h. The coated plates were then washed with 300 μl of wash buffer (0.05% Tween-20 in PBS) for 5 times by means of an automatic plate washer (Tecan HydroSpeed™ plate washer). Then they were blocked by adding 100 μl/well of blocking buffer (10% horse serum and 5% skimmed milk in PBS) for 30 min at 37 °C, then, the blocking buffer was discarded. The samples were diluted (1: 40, based on the result from checkerboard analysis) in dilution buffer (10% horse serum, 5% skimmed milk, and 10% *E. coli* lysate in PBS). The 100 μl/well of diluted samples were added to the blocked plates and incubated for 30 min at 37 °C. Then, five-time washing was done as described above. Afterward, 100 μl/well of 1: 25 (based on the result of checkerboard analysis) diluted rabbit anti-goat IgG (H + L) in secondary antibody buffer was added to the well and incubated for 30 min at 37 °C. Thereafter, 5 times of washing procedure as described were done. 100 μl of KPL TMB microwell peroxidase substrate (SureBlue™ TMB 1-Component Microwell Peroxidase Substrate) was added to each well and then incubated for 15 min at room temperature. The reaction was stopped by means of 100 μl of the stop solution (0.25 M HCl). The optical density (O.D.) was measured at 450 nm.

### ELISA optimization

To define the optimal concentration of recombinant protein for coating on the wells, the titration checkerboard was performed. The concentration of the purified antigen was 0.5 mg/ml. The plates were coated with 100 μl of two-fold serial dilution of recombinant gag protein and incubated for 1 h at 37 °C. Washing of the coated plates was performed 5 times using wash buffer. Then, the blocking buffer (100 μl) was added to each well for 30 min at 37 °C. The two-fold serial diluted serum samples in dilution buffer were added to the blocked plates and incubated for 30 min at 37 °C. Then, the general protocol of ELISA was done as described above. The 334 negative samples and 31 positive samples were tested, and the results were used for sensitivity and specificity calculation.

### Statistical analyses

Statistical calculations were done in Stata statistical software (Release 16, StataCorp, College Station, TX). The optimal cut off value, sensitivity, and specificity for in-house ELISA were established. The expected test performance was evaluated using the receiver operating characteristic (ROC) curve. The area under the ROC curve was obtained for accuracy evaluation. The degree of agreement between the results of commercial ELISA test kit (IDEXX CAEV/MVV total Ab test®, IDEXX Laboratories, Inc., Maine, USA) and developed in-house ELISA was measured using Cohen’s Kappa statistic, which the degree of agreement was interpreted according to Landis and Koch^28^.

### Ethics approval

The experimental protocol of this study was conducted under the ethical approval of Kasetsart University’s Institutional Animal Care and Use Committee. The approved number is ACKU64-VET-007. The authors confirm that the ethical policies of the journal, as noted on the journal’s author guidelines page, have been adhered to and the appropriate ethical review committee approval has been received. The study was also reported according to the ARRIVE guidelines.

## Results

### Phylogenetic tree construction of SRLVs based on* gag* fragment

**Figure 1:**
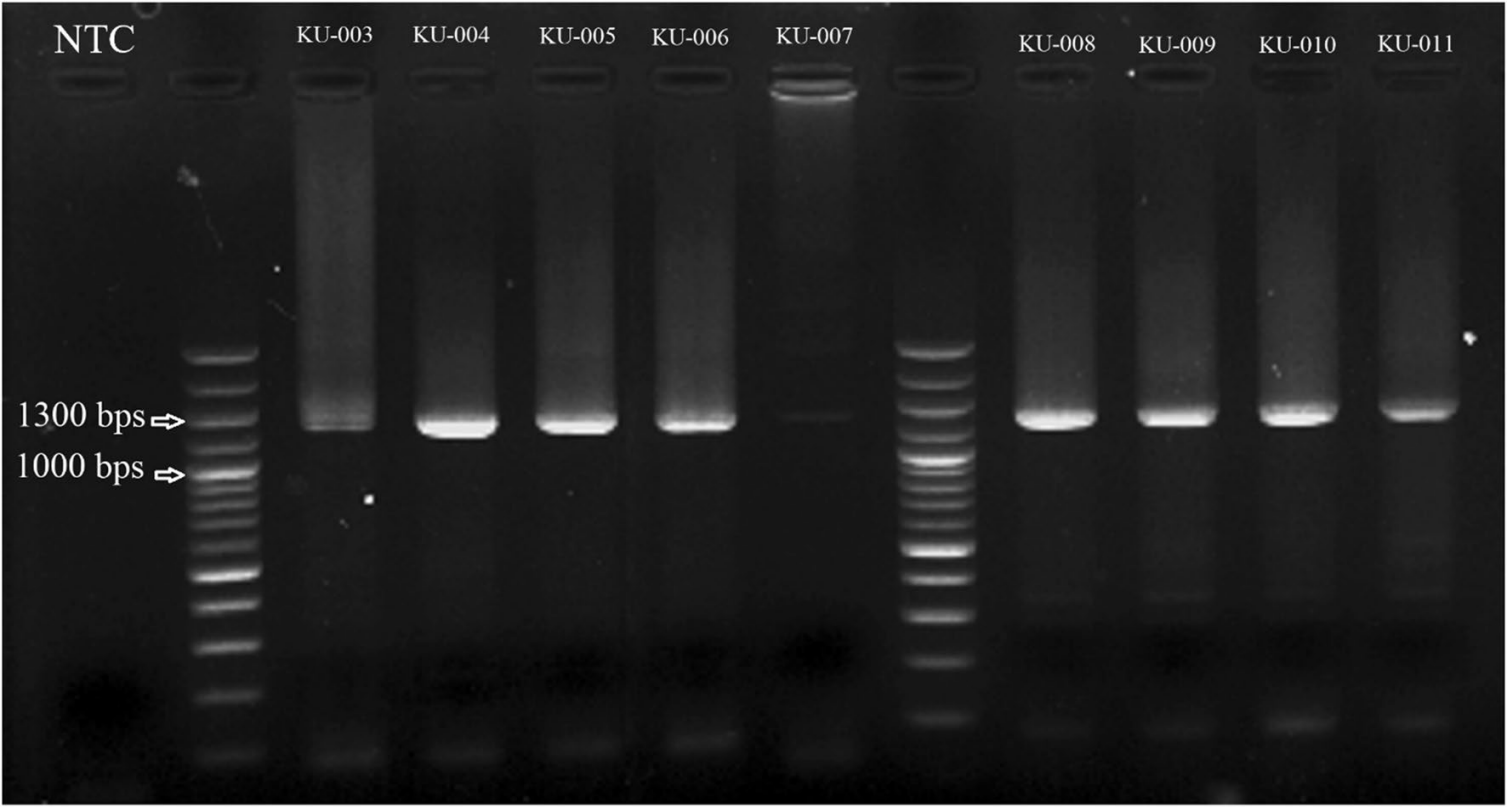
Result of nested PCR of shows the band of viral nucleic acid which the target size is ~ 1300 bps. NTC stand for no template control.

**Figure 2:**
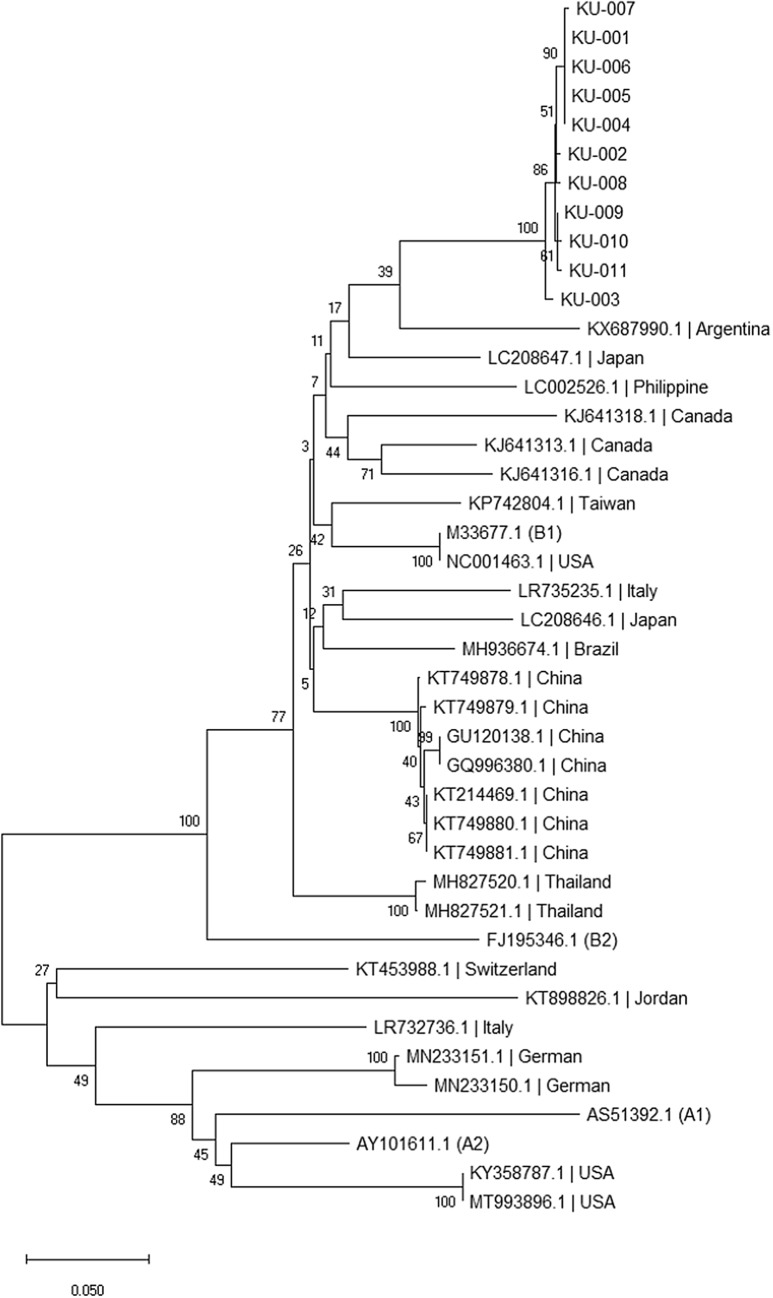
Neighbor-Joining (NJ) phylogenetic tree based on partial *gag* gene sequences showing genetic relationship between the Thai SRLV isolates obtained in this study and other strains. The analysis involved a total of 550 base pairs from 40 nucleotide sequences. The sequences reported in this study were different from Thai sequences reported in 2018 (MH827520.1, MH827521.1). The numbers displayed on the node represent the percentage of bootstrap values obtained from 1000 replicates. The lengths of the branches indicate the number of substitutions per site. The nucleotide sequences exhibit similarities ranging from 84.81% to 88.76% compared to the reference sequence (MH827520.1) which was previously reported in Thailand. The sequences found in this study are similar to B1 group which differs from the previous report that closer to B2 group.

The nested PCR of the partial *gag* gene was carried out. The results of nested PCR were shown in Fig. 1. There were 11 samples showed positive results and all positive samples were sequenced. The DNA obtained from KU-002 was isolated from brain tissue whereas the DNA from KU-001, KU-003 to KU-011 were extracted from spleen. The sequences of SRLV isolates were analyzed in comparison with reference sequences retrieved from databases. The strain of SRLVs circulating in Thailand is B2. A phylogenetic tree was shown in Fig. 2. The phylogenetic tree showed the sequences reported in this study are different from the Thai sequences reported previously. The similarities of nucleotide sequences range between 84.81% and 88.76%, whereas the similarities of amino acid sequences are 82.01–88.65% in comparison to MH827520.1 which was reported in 2018.

### Western blot analysis

**Figure 3:**
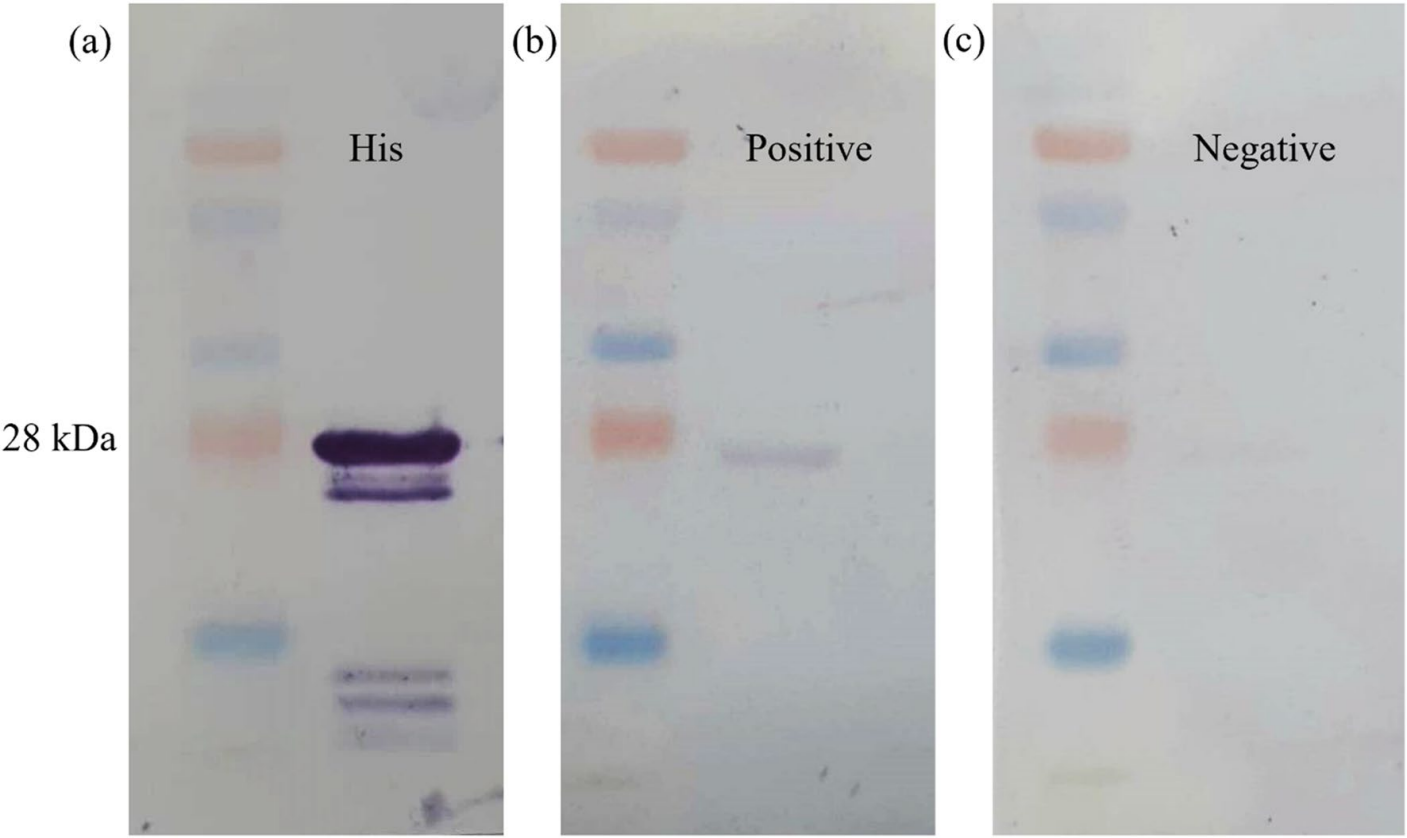
Results of western blotting assay of recombinant gag protein of SRLVs; (**a**) displayed a specific band upon utilizing the anti-histidine antibody as the primary antibody, indicating the protein expression is not inhibited. (**b**) a specific band was observed when confirmed positive serum was used as the primary antibody. This indicated the capacity of the natural antibody to effectively bind to the recombinant gag protein. (**c**) the negative result when negative serum was used as the primary antibody.

The western blotting was achieved for the detection of an immunological reaction between natural antibodies and recombinant protein which the size around 28 kDa. The results were shown in Fig. 3. A positive serum sample was able to bind the recombinant gag protein while a negative serum sample was unable to bind the protein.

### ELISA optimization and evaluation

#### Checkerboard analysis result

Eight concentrations of the antigen were tested. Two-fold dilutions of 0.5 mg/ml of antigen were tested. The optimal coating concentration was 12.5 μg/ml. While the two-fold dilutions of the detecting antibody were tested. The suitable dilution of the detecting antibody was 1:25 in the primary antibody buffer.

#### Statistical analysis result

**Figure 4:**
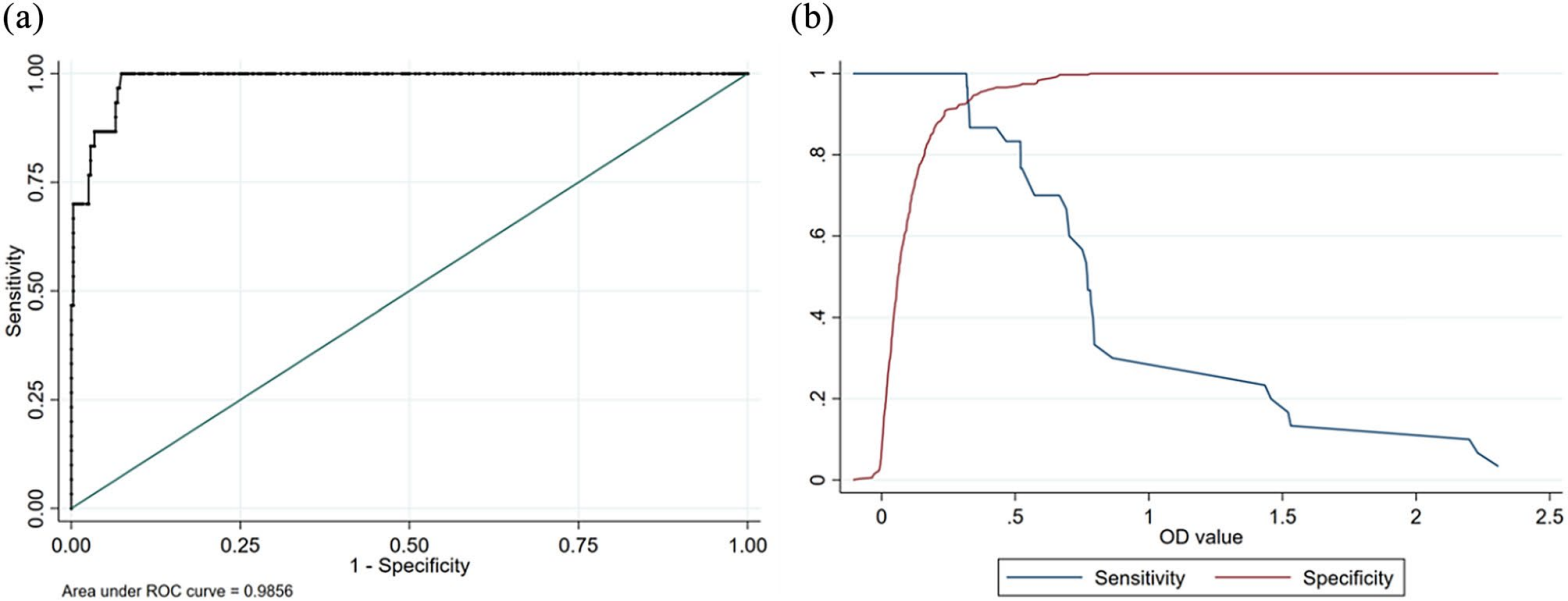
(**a**) The receiver operating characteristic (ROC) curve with area under the curve, (**b**) the relationship between sensitivity, specificity and O.D.

The receiver operating characteristic (ROC) curve with area under the curve (0.9856) is shown in Fig. 4. The developed in-house ELISA can be considered as “excellent” according to the AUC^29^. The optimal cut off value is 0.321 with 96.67% sensitivity and 93.18% specificity. The Cohen’s Kappa statistic, is 0.66 (95% confidence interval = 0.55–0.78), indicated that developed in-house ELISA showed “substantial” agreement with commercial ELISA test kit (IDEXX CAEV/MVV total Ab test®, IDEXX Laboratories, Inc., Maine, USA).

## Discussion

Small ruminant lentiviruses (SRLVs) are known to induce chronic progressive inflammation in small ruminants. Despite posing a significant challenge due to the lack of an effective vaccine and specific treatment, disease control heavily relies on diagnosis. Nowadays, the gold standard for SRLV infection diagnosis remains undetermined^30^. However, the serological investigation could decrease the prevalence of SRLV infection in goats^15,31,32^. The ELISAs are appropriate for the large numbers of samples screening. The antigen detection methods such as conventional PCR, nested PCR, or qPCR have been designed to detect SRLVs in seronegative animals. However, the low quantity of viral load among infected animals and the antigenic variation of the virus can lead to failure of either antigen or antibody detection. As shown in the phylogenetic tree (Fig. 2), the sequences of partial *gag* gene of the virus reported in this study were different from Thai sequences reported previously in 2018. Since the high mutation rate of retroviruses, the phylogenetic data should be employed to select the proper epitopes from the circulating genotypes of the virus for use in ELISAs. The gag protein is the choice protein for the development of immunoassays for antibody detection due to its conservative and antigenic properties of this protein^33,34^. The recombinant gag polypeptide produced in this study was expressed in the soluble fraction. The hydrophilic property causes the polypeptide to be easily recoverable from the culture system without denaturing agents. Absence of denaturing agents while purification processes could reduce the misfolding of the polypeptide which may cause the false negative results.

Use of the combination of different genotypes of SRLV in the same ELISA well can improve the detection throughout the course of infection, as compared to using of a commercial ELISA test kit^35^. The use of combination recombinant protein derived from various strains within the same well can improve the sensitivity of ELISA. This was attributed to their capability to detect diverse strain of virus. In this study, two genotypes of the partial *gag* gene of SRLVs were selected and coated into ELISA wells for reducing the false negative result. This notion was based on the identification of the most hydrophobic segment within the *gag* gene from a total of 11 samples, which were subsequently categorized into two distinct groups. The result from the ELISA test developed using recombinant gag protein achieved a specificity and sensitivity of 93.18% and 96.67%, respectively, compared to a commercial test kit. This indicates that the test could be effectively applied for SRLV infection diagnosis in goats.

## Conclusions

The phylogenetic analysis of SRLVs shows that the circulating viruses found in this study have significantly changed their nucleic acid sequences from the previous report. The indirect ELISA developed with the partial part of gag protein based on circulating strain of SRLVs as an antigen for detection of anti-SRLV IgG has high sensitivity (96.67%) and specificity (93.18%).

## Data Availability

All data collected and analyzed in this research are included in the article.
